# Expression profiles of glucocorticoid-inducible proteins in human papilloma virus-related oropharyngeal squamous cell carcinoma

**DOI:** 10.3389/froh.2023.1285139

**Published:** 2023-10-25

**Authors:** Mahmood S. Mozaffari, Rafik Abdelsayed, Sahar Emami, Sravan Kavuri

**Affiliations:** ^1^ Department of Oral Biology and Diagnostic Sciences, The Dental College of Georgia, Augusta University, Augusta, GA, United States; ^2^ Department of Pathology, Medical College of Georgia, Augusta University, Augusta, GA, United States

**Keywords:** human papilloma virus, oropharyngeal, squamous cell carcinoma, GILZ, Annexin-A1, SGK-1, glucocorticoids

## Abstract

**Introduction:**

Human papillomavirus virus-related oropharyngeal squamous cell carcinoma (HPV-OPSCC) comprises a significant portion of head and neck cancers. Several glucocorticoid-inducible proteins play important roles in pathogenesis of some cancers but their status and roles in HPV-OPSCC remain elusive; these include the glucocorticoid-induced leucine zipper (GILZ), Annexin-A1 and serum glucocorticoid-regulated kinase-1 (SGK-1).

**Methods:**

We determined expression profiles of these proteins, using immunohistochemistry, in archived biopsy samples of patients diagnosed with HPV-OPSCC; samples of non-cancer oral lesions (e.g., hyperkeratosis) were used as controls.

**Results:**

GILZ staining was primarily confined to nuclei of all tissues but, in HPV-OPSCC specimens, neoplastic cells exhibiting mitosis displayed prominent cytoplasmic GILZ expression. On the other hand, nuclear, cytoplasmic and membranous Annexin-A1 staining was observed in suprabasal cell layers of control specimens. A noted feature of the HPV-OPSCC specimens was few clusters of matured and differentiated nonbasaloid cells that showed prominent nuclear and cytoplasmic Annexin-A1 staining while the remainder of the tumor mass was devoid of staining. Cytoplasmic and nuclear staining for SGK-1 was prominent for control than PV-OPSCC specimens while staining for phosphorylated SGK-1 (pSGK-1; active) was prominent for cell membrane and cytoplasm of control specimens but HPV-OPSCC specimens showed mild and patchy nuclear and cytoplasmic staining. Semi-quantitative analysis of GILZ immunostaining indicated increased staining area but similar normalized staining for HPV-OPSCC compared to control specimens. By contrast, staining area and normalized staining were reduced for other proteins in HPV-OPSCC than control specimens.

**Discussion:**

Our collective observations suggest differential cellular localization and expression of glucocorticoid-inducible proteins in HPV-OPSCC suggestive of different functional roles in pathogenesis of this condition.

## Introduction

Cancers of the head and neck are the sixth and eighth most common types of cancers in the world and the United States, respectively. Greater than 90% of head and neck cancers are squamous cell carcinomas that develop from the mucosal epithelium of the oral cavity, sinonasal tract, pharynx and larynx (1–3). Tobacco-derived carcinogens, excessive intake of alcohol, or both, are major contributors to the burden of head and neck squamous cell carcinoma; these tumors are commonly found on the anterior 2/3 of the tongue, floor of the mouth, palate, buccal mucosa, sulcus and gingiva (1, 2). Importantly, oropharyngeal squamous cell carcinoma (OPSCC) is linked to prior infection with oncogenic strains of the human papilloma virus (HPV), with HPV-16 as the major culprit; the oncoproteins E6 and E7, via promoting degradation of tumor suppressor proteins, p53 and retinoblastoma, respectively, are responsible for cell cycle dysregulation and malignant transformation of HPV-infected epithelial cells (4). These tumors are found on oropharynx, tonsillar region, base of the tongue, soft palate and uvula of younger and mostly male (about 75%) patients who often have lymph node metastasis at diagnosis (4). While the incidence of both HPV-positive and HPV-negative OPSCC has increased over the last 20 years, the HPV-positive variety is increasing much more rapidly despite the hope that vaccination campaign would curb it (5, 6). Indeed, while the incidence of HPV-positive cervical cancer has declined in both the United States and the United Kingdom (1995–2015), the incidence of HPV-positive OPSCC has increased for both countries. Interestingly, patients with HPV (16)-positive OPSCC have longer overall survival and a lower rate of recurrence after treatment than those with HPV-negative OPSCC (4–6). Treatment modalities for OPSCC include surgery and chemoradiation with greater utilization of minimally-invasive surgical approaches for HPV-positive cases (6). Importantly, given the increasing recognition of the susceptibility of HPV-positive tumors to antitumor immunity (4–6), there is intense interest in better understanding of the tumor microenvironment for eventual development of novel immunotherapies.

Aside from the use of glucocorticoids for a variety of non-cancer disorders, they are also used for treatment of lymphohematopoietic neoplasms and as adjuvant therapy in non-hematological malignancies in order to limit adverse effects of chemotherapy and/or radiotherapy (7). Importantly, the following aspects are of relevance in relation to glucocorticoids and HPV-OPSCC. First, aside from the adrenal glands as the primary source of endogenous glucocorticoids, steroid metabolism occurs in other tissues including the oral mucosa (8). Second, glucocorticoids are well-known regulators of immune and inflammatory responses and, thus, could impact the tumor microenvironment (9). This is consistent with intense interest in unraveling of the immune infiltrative landscape of HPV-positive vs. -negative head and neck tumors which, in turn, would help to better understand more favorable clinical outcome of HPV-positive than -negative squamous cell carcinoma and also strengthen the rationale for immunotherapy modalities (10–12). Third, glucocorticoids can affect the outcome of epithelial tumors via a number of mechanisms including impaired immune surveillance, anti-apoptotic effects, increased tumor cell energy metabolism and promoting chemotherapy resistance (7). Fourth, glucocorticoids regulate expression of several proteins which, aside from regulating immune and anti-inflammatory responses of glucocorticoids, they are implicated in etiopathogenesis of various cancers; these include the glucocorticoid-induced leucine zipper (GILZ), serum glucocorticoid-regulated kinase-1 (SGK-1) and Annexin-A1 (ANXA1) (13–15). Indeed, we recently showed differential cellular localization of GILZ, SGK-1 and phosphorylated (active) form of SGK-1 (pSGK-1) in potentially malignant and malignant human oral lesions (i.e., HPV-negative) (7). However, the status of glucocorticoid-inducible proteins in HPV-OPSCC remain to be established. Thus, the present study tested the hypothesis that expression profiles of glucocorticoid-inducible proteins are different in HPV-OPSCC lesions than those of non-neoplastic oral mucosa.

## Materials and methods

**Figure 1:**
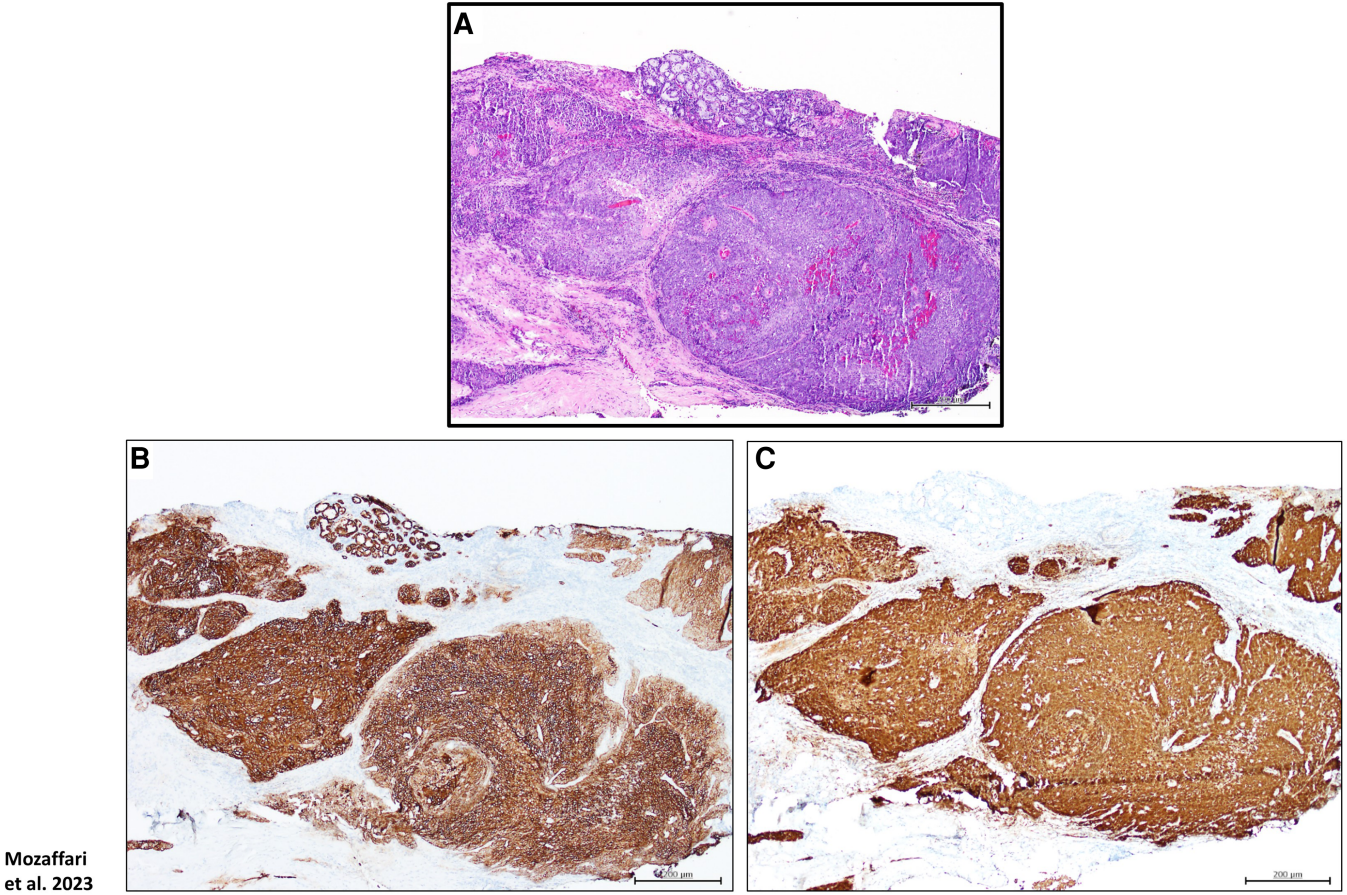
Panels show H&E stain (**A**), p16 (**B**) as well as AE1/AE3 (**C**) immunostaining for a HPV-OPSCC tissue specimen; these protocols were used for final diagnosis of cases clinically suspected of HPV-OPSCC. Scale bar: 200 μm.

The archived tissues in the department of pathology at the Augusta University-Medical Center and the diagnostic pathology laboratory at the Dental College of Georgia were searched for cases with diagnosis of HPV-related oropharyngeal carcinoma (HPV-OPSCC; *n* = 8) as well as benign keratinized lesions which served as non-neoplastic control cases (*n* = 5); this retrospective study was considered exempt from review by the Institutional Review Board. The HPV-OPSCC cases were initially diagnosed based on clinical examination, histopathological features of biopsy specimens [using hematoxylin-eosin (H&E) stain] as well as immunostaining for p16 and epithelial markers- AE1/AE3 (Figure 1). Table 1 shows demographic information, site of lesion and initial clinical impression/diagnosis (i.e., prior to biopsy, histopathological examination and immunostaining).

**Table 1:** Demographic information, site of lesion and clinical impression/diagnosis of subjects whose tissue specimens were used in this study.

Age (Years)	Sex	Ethnicity	Anatomical site	Initial clinical impression/diagnosis
Control subjects				
71	Male	Caucasian	Right retromolar pad	Leukoplakia; Benign keratosis
61	Female	Caucasian	Right buccal mucosa	Leukoplakia
66	Male	African-American	Junction of hard and soft palate	Leukoplakia; frictional hyperkeratosis
62	Male	Caucasian	Mandibular gingiva	Frictional keratosis
38	Male	African-American	Mandibular gingiva	Hyperkeratosis
Subjects with HPV-Positive OPSCC				
68	Male	Caucasian	Base of tongue	HPV-positive SCC
70	Male	Caucasian	Oropharynx	HPV-positive SCC
67	Male	Caucasian	Oropharynx	HPV-positive SCC
55	Male	Caucasian	Base of tongue	HPV-positive SCC
76	Male	Caucasian	Base of tongue	HPV-positive SCC
68	Male	Caucasian	Mandibular retromolar pad	Dysplasia; SCC
67	Male	Caucasian	Soft palate	Traumatic ulcer; SCC; deep fungal infection
60	Male	African-American	Tonsillar pillar	Lymphoma; SCC; Neoplasm

SCC, Squamous Cell Carcinoma.

The corresponding paraffin-embedded tissue blocks, of the aforementioned cases, were identified and 5 μm tissue sections were cut from each tissue block of the HPV-OPSCC and benign hyperkeratosis control cases followed by mounting on glass slides. Tissue sections were deparaffinized in a Leica Auto-Stainer XL and antigen retrieval was performed using a Citric Acid based Antigen Unmasking Solution (Vector Laboratories, Burlingame, CA). Thereafter, tissue sections were treated with 0.3% hydrogen peroxide for 30 min. at room temperature, washed in water and then incubated with Blocking solution (2.5% horse serum, 1% BSA, 0.5% Triton X-100) for at least 1 hour at room temperature. Each primary antibody was diluted (1:100) in Blocking solution and incubated with tissue sections overnight at room temperature. GILZ and SGK-1 polyclonal antibodies were procured from ABclonal (Catalog numbers A6779 and A1025, respectively), pSGK-1 polyclonal antibody (Ser422) was obtained from Invitrogen (Catalog number 44-1264G) and Annexin-A1/ANXA1 (ab214486) was purchased from Abcam. Tissue sections were then washed twice in PBS and incubated for 1 h at room temperature with horseradish peroxidase-conjugated secondary antibody (Vector Laboratories, Burlingame, CA) followed by 3,3'-diaminobenzidine (DAB) staining using the ImmPACT DAB Substrate Kit (Vector Laboratories, Burlingame, CA). Slides were counterstained with hematoxylin and mounted using mounting medium. To establish effectiveness of each antibody for the targeted protein, human tonsillar tissue was used for Annexin-A1 while human mammary tissue was used to establish staining for each of the other proteins of interest. Negative controls were performed on additional tissue sections which excluded the primary antibodies.

The Image J Fiji software was used for semi-quantitative analyses of immunohistochemical staining following a previously described protocol (16, 7). This protocol involves deconvolution of immunohistochemistry images followed by the assessment of DAB staining, using mean gray intensity, and normalization to the nucleus. Further, we measured the fractional area of staining.

### Statistics

Semi-quantitative data are reported as means ± SEM. Data for each protein of interest were analyzed using unpaired t-test to establish significance (*p* < 0.05) between non-neoplastic control and HPV-OPSCC specimens.

## Results

### Hematoxylin and eosin-stained sections

**Figure 2:**
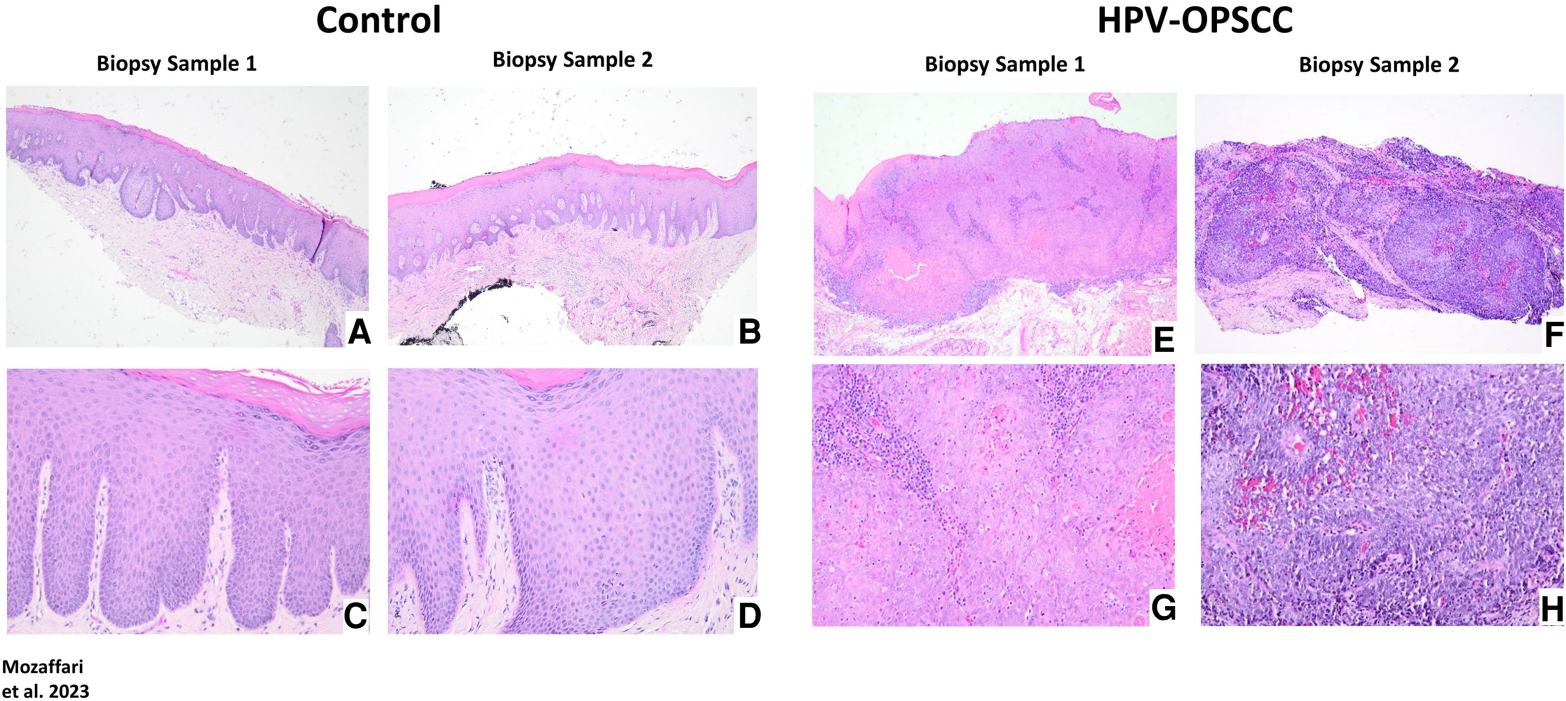
Panels show representative H&E images for control and HPV-positive OPSCC tissue samples. 40x: (**A**–**B**), (**E**–**F**); 100x: (**C**–**D**), (**G**–**H**).

The non-neoplastic control specimens showed variably-thickened surface epithelium covered by predominantly thickened layer of orthokeratin and focally by parakeratin. The spinous cell layer of the epithelium is composed of polyhedral cells exhibiting uniform basophilic nuclei surrounded by abundant eosinophilic cytoplasm and maintained the usual pattern of cellular maturation (Figures 2A–D). The HPV-OPSCC specimens shared common histopathologic features of predominantly invasive non-keratinizing neoplastic epithelial proliferation supported by connective tissue stroma with surface epithelium exhibiting variable degrees of epithelial dysplasia (Figures 2E–H). The neoplastic epithelial proliferation is present in the form of variably-sized islands and sheets of basaloid cells. The cells exhibited pleomorphic and hyperchromatic nuclei surrounded by scant cytoplasm, variable numbers of mitotic figures and patchy areas of necrosis. Minimal keratinization, less than 10% of tumor volume, was noted in some HPV-OPSCC cases.

### GILZ immunostaining

**Figure 3:**
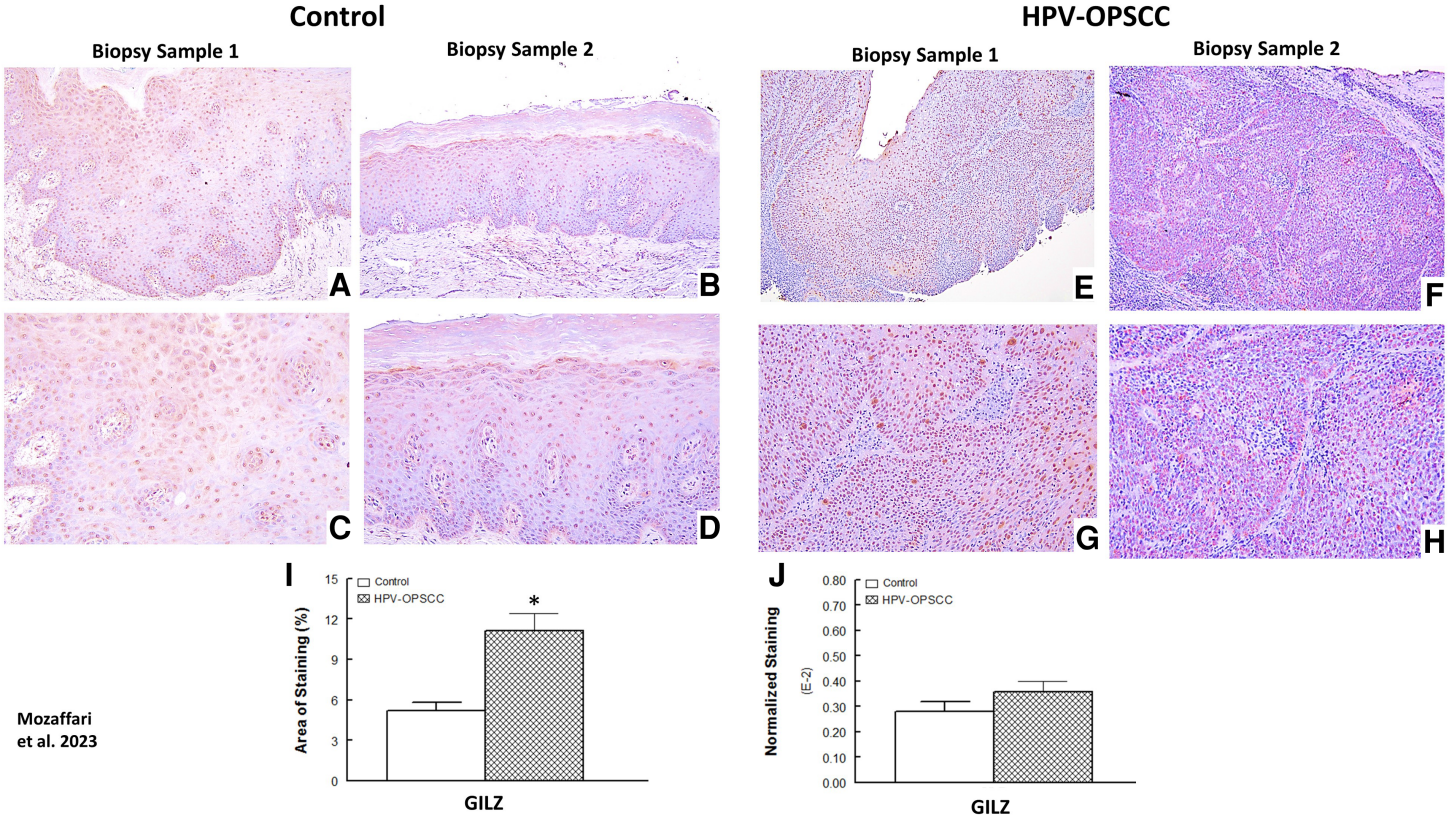
Panels show representative images for GILZ staining for control and HPV-OPSCC tissue samples. GILZ is primarily localized to nuclei all specimens; however, cytoplasmic staining is also evident for mitotically active cells in HPV-OPSCC specimens. Also shown are percent area of staining and normalized staining for both conditions (panels (**I**) and (**J**), respectively). 40x: (**A**–**B**), (**E**–**F**); 100x: (**C**–**D**), (**G**–**H**). * *p* < 0.05 compared to the control group.

**Figure 4:**
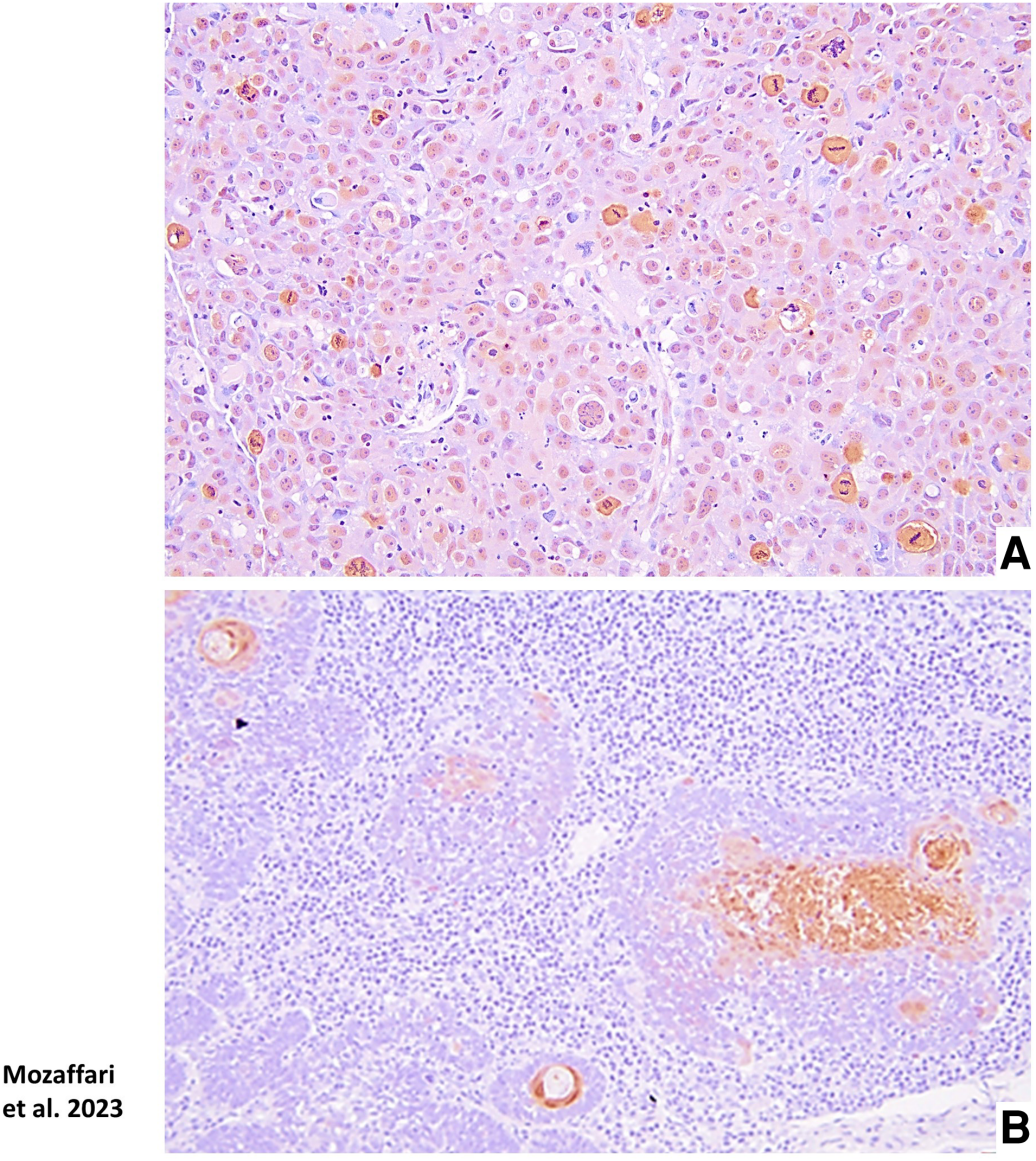
Panel (**A**) shows cytoplasmic GILZ expression surrounding mitotic figures, some of which are atypical, in a HPV-OPSCC specimen. Panel (**B**) shows Annexin-A1 staining confined to matured, differentiated non-basaloid portions of a HPV-OPSCC specimen.

The benign hyperkeratosis control tissue specimens showed prominent nuclear staining for GILZ (Figures 3A–D). HPV-OPSCC sections showed prominent uniformly distributed nuclear staining in the majority of neoplastic basaloid non-keratinized epithelial cells in all cases (Figures 3E–H). It is noteworthy that neoplastic cells exhibiting mitotic figures in HPV-OPSCC cases demonstrated cytoplasmic GILZ staining surrounding mitotic figures some of which were atypical, a feature very prominent for some tissue specimens (Figure 4A). Semi-quantitative analyses indicated significant increase in GILZ staining area but similar normalized staining (Figures 3I–J), the latter related to greater number of nuclei in HPV-OPSCC cases.

### Annexin-A1 immunostaining

**Figure 5:**
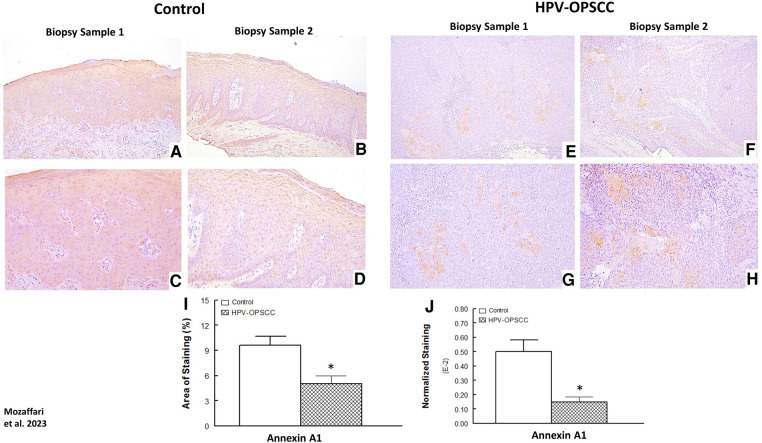
Panels show representative images for annexin-A1 staining for control and HPV-OPSCC tissue samples. Annexin-A1 staining was observed for suprabasal cell layers of control specimens while staining was confined to differentiated non-basaloid neoplastic cells in HPV-OPSCC specimens. Also shown are percent area of staining and normalized staining for both conditions (panels (**I**) and (**J**), respectively). 40x: (**A**–**B**), (**E**–**F**); 100x: (**C**–**D**), (**G**–**H**).

The benign hyperkeratosis specimens displayed nuclear, cytoplasmic and membranous staining for Annexin-A1 in the suprabasal epithelial cells (Figures 5A–D). The intensity of staining was generally uniform throughout the suprabasal cells. The HPV-OPSCC specimens showed prominent nuclear and cytoplasmic staining predominantly confined to patchy areas where matured and differentiated non-basaloid neoplastic cells were seen; however, the majority of basaloid neoplastic epithelial cells lacked Annexin-A1 expression (Figures 5E–H; Figure 4B). Semi-quantitative analyses indicated significantly lower staining area and normalized staining for HPV-OPSCC than control cases (Figures 5I–J).

### SGK-1 and pSGK-1 immunostaining

**Figure 6:**
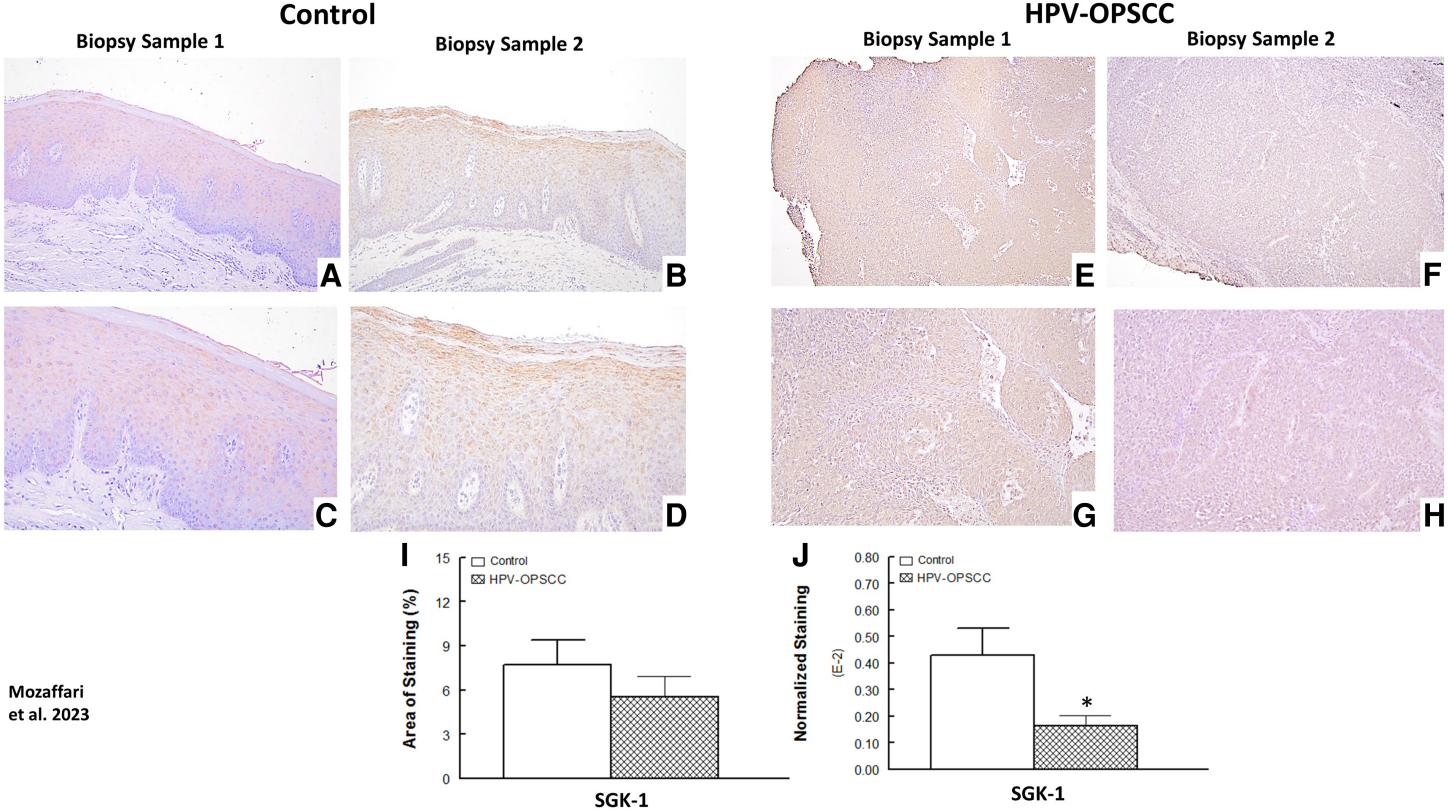
Panels show representative images for SGK-1 staining for control and HPV-OPSCC tissue samples. Compared to HPV-OPSCC specimens, control tissue samples showed prominent cytoplasmic and nuclear staining for SGK-1. Also shown are percent area of staining and normalized staining for experimental groups (panels (**I**) and (**J**), respectively). 40x: (**A**–**B**), (**E**–**F**); 100x: (**C**–**D**), (**G**–**H**). * *p* < 0.05 compared to the control group.

The benign hyperkeratosis cases showed nuclear and perinuclear cytoplasmic staining**;** the staining intensity increased gradually from the suprabasal layers of cells to the higher superficial layers of cells within the surface epithelium (Figures 6A–D). On the other hand, the HPV-OPSCC specimens showed heterogeneous and less clearly-defined SGK-1 immunostaining (Figures 6E–H). Semi-quantitative analyses indicated similar staining area between the two conditions but reduced normalized staining in the HPV-OPSCC than control cases (Figures 6I–J), the latter related to greater number of nuclei in OPSCC cases.

**Figure 7:**
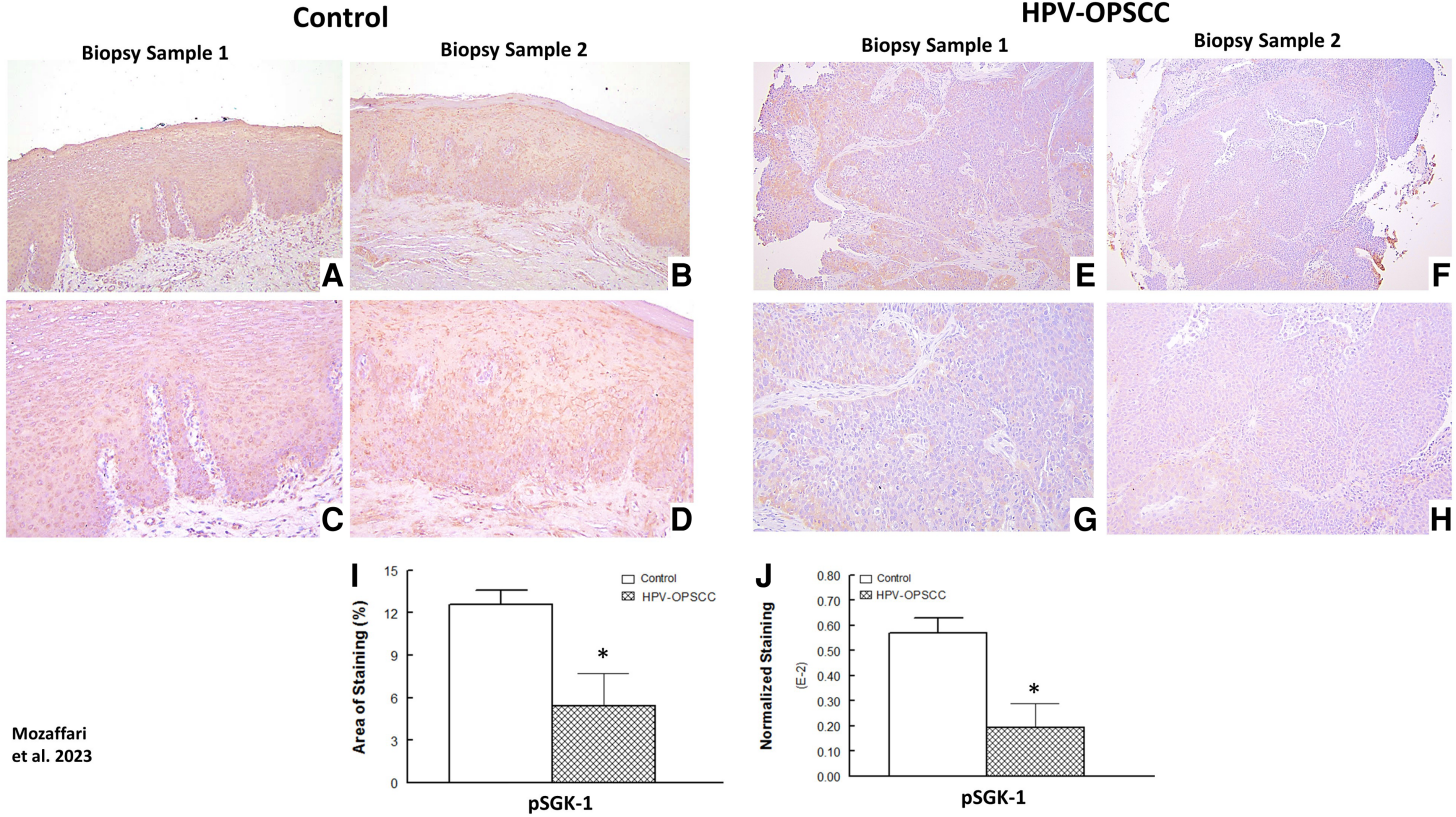
Panels show representative images for SGK-1 staining for control and HPV-OPSCC tissue samples. Control tissue specimens, displayed prominent cytoplasmic and plasma membrane pSGK-1 staining while HPV-positive OPSCC specimens showed mild and patchy cytoplasmic and nuclear staining. Also shown are percent area of staining and normalized staining for experimental groups (panels (**I**) and (**J**), respectively). 40x: (**A**–**B**), (**E**–**F**); 100x: (**C**–**D**), (**G**–**H**). * *p* < 0.05 compared to the control group.

The benign hyperkeratosis cases showed primarily cytoplasmic and membranous staining for pSGK-1 while the HPV-OPSCC cases showed mild and patchy nuclear and cytoplasmic staining (Figures 7A–D vs. E–H). Semi-quantitative analyses revealed significantly reduced area of staining and normalized staining for HPV-OPSCC compared to control cases (Figures 7I–J).

## Discussion

The present study shows differential expression and cellular localization of several glucocorticoid-inducible proteins in HPV-OPSCC. First, while GILZ staining was mainly confined to nuclei of all specimens, prominent cytoplasmic GILZ staining was observed for mitotically active cells in HPV-OPSCC. Second, Annexin-A1staining was observed for cytoplasm, plasma membrane and nuclei of suprabasal cell layers of control specimens while, in the HPV-positive OPSCC specimens, Annexin-A1 staining was confined to nuclear and cytoplasm of clusters of, matured and differentiated, non-basaloid neoplastic cells within the tumor mass. Third, compared to HPV-OPSCC specimens, control tissue samples showed prominent cytoplasmic and nuclear staining for SGK-1. Fourth, control tissue specimens, displayed prominent cytoplasmic and plasma membrane pSGK-1 staining while HPV-positive OPSCC specimens showed mild and patchy cytoplasmic and nuclear staining. Fifth, semi-quantitative analyses indicated significant increase in the staining area, but not normalized staining, for GILZ in HPV-OPSCC specimens compared to control tissues; however, staining area and normalized staining were reduced for other proteins of interest in tumor specimens compared to non-neoplastic, hyperkeratotic, oral tissue samples. Collectively, our observations are suggestive of differential functional roles for glucocorticoid-inducible proteins in HPV-OPSCC.

GILZ is a well-established mediator of immune and inflammatory responses (17) and, thus, is of major relevance in relation to cancer; however, its impact in cancer is cell- and context-specific (18) as exemplified by the following. First, upregulation of GILZ in dendritic cells, of the tumor microenvironment, suppressed the T cell response against cancer (19). Second, GILZ- is implicated in dexamethasone-induced antiproliferative effect on activated T lymphocytes (20). Third, proliferative and oncogenic activities are attributed to GILZ for ovarian cancer and melanoma and resistance to cyclopamine therapy in lung cancer is attributed to high GILZ expression (21–23). Fourth, L-GILZ (a variant of GILZ) regulated tumor growth via facilitation of p53 function as a tumor suppressor protein (24). Fifth, overexpression of L-GILZ in human thyroid cell line inhibited cell growth both *in vitro* and in xenograft transplantation in mice (13, 25, 26). In a recent study, we observed nuclear GILZ staining for dysplastic, oral squamous cell carcinoma (OSCC; HPV negative) and benign keratosis specimens (7). While GILZ expression was higher in epithelial dysplasia, compared to benign keratosis, its level in OSCC was reduced to that of benign keratosis (7). Our observation of prominent nuclear staining for GILZ, in this study, is consistent with our aforementioned investigation. Nonetheless, two features of HPV-OPSCC (compared to OSCC vs. benign keratosis) are noteworthy. First, while staining area was significantly increased in HPV-OPSCC, compared to hyperkeratosis, normalized staining was similar between the two groups, an aspect related to greater number of nuclei in HPV-OPSCC. Second, marked cytoplasmic staining for GILZ in mitotically active cells of HPV-OPSCC specimens is suggestive of its possible involvement in cell division. To our knowledge, a finding similar to the latter observation has not been reported previously. Thus, whether GILZ plays a role in cell division or it is a feature unique to HPV-OPSCC remains to be established; elucidation of these aspects could be of translational value with respect to tumor progression and/or delineation of HPV positive vs. HPV negative tumors. Nonetheless, it is noteworthy that a recent study suggests that L-GILZ is an important factor for undifferentiated spermatogonia and spermatogenesis (27).

Annexin-A1 was initially discovered as a protein mediating potent anti-inflammatory effects of glucocorticoids (28). Increasingly, however, multiple other functions are attributed to it including its role in cancer although its regulation of tumor growth and metastasis remains to be resolved. Nonetheless, while Annexin-A1 expression is reportedly increased in some tumors (e.g., gut, esophagus, lung, etc.), its expression is seemingly lost in other tumors such as head and neck squamous cell carcinoma (HNSCC) (14). Indeed, Annexin-A1 down regulation in HNSCC was associated with poor histological differentiation, advanced T stages, hypopharyngeal localization and lymphoregional lymph node metastasis (29). Further, dysplastic epithelium also showed significant loss of Annexin-A1 compared to normal epithelium. On the other hand, well-differentiated tumors displayed positive Annexin-A1 signals in highly keratinized segments while poorly differentiated tumors exhibited very weak or negative staining (29). Authors suggested that loss of Annexin-A1 is a consequence of rather than an etiological factor for tumor development. Others have reported markedly reduced staining for Annexin-A1 in tissue specimens diagnosed with nasopharyngeal carcinoma compared to noncancerous mucosa of same site (30). Further, Annexin-A1 downregulation was related to squamous differentiation status of these tumors as revealed by the observation of positive Annexin-A1 signal in differentiated areas while poorly differentiated tumors displayed negative staining. Similarly, reduced Annexin-A1 expression is reported in oral squamous cell carcinoma and that its expression negatively correlated with the pathologic differentiation grades of tumors (31). However, in these studies the status of the tumor in relation to HPV is not clear. Using proteome analyses, downregulation of Annexin-A1 is reported in HPV-positive HNSCC while its expression is upregulated in HPV-negative tumors (32). Authors postulated that differential expression of Annexin-A1, as a function of HPV status, may reflect attempts to regulate expression of pro-inflammatory cytokines (e.g., IL-6) in tumor cells. In agreement with previous studies, we also observed significant reduction in Annexin-A1 staining in HPV-OPSCC specimens compared to control tissues; nonetheless, discernable Annexin-A1 staining of OPSCC specimens was confined to few clusters of matured, differentiated non-basaloid neoplastic cells (i.e., keratin forming) within the tumor mass. It is noteworthy that while HPV-OPSCC is a non-keratinizing form of squamous cell carcinoma, some foci of keratin-forming tumor cells can be found in these tumors. Thus, in terms of translational value, Annexin-A1 expression may help delineate HPV-negative keratin-forming vs. HPV-positive non-keratinizing forms of squamous cell carcinoma.

SGK-1, a member of the family of serine-threonine kinases, signals downstream of the phosphatidylinositol 3- kinase and shares similar structure, substrate specificity and function with Akt (33). Similar to Akt, phosphorylation of SGK-1 results in its activation and pro-survival activity (34, 35). Increasingly, important roles for SGK-1 have been proposed in cancer biology (15). For example, increased expression and/or activity of SGK-1 is reported for several human tumors including those involving the breast, tongue, head and neck (e.g., squamous cell carcinoma), ovarian, prostate, multiple myeloma and non-small cell lung cancer (36–43). Further, increased expression and/or activation of SGK-1 contribute to invasiveness of tumors, metastasis and treatment resistance (44–46, 37). Thus, SGK-1 has emerged as an important therapeutic target and its inhibition shown to exert beneficial effects in inducing cell death and potentiating susceptibility to chemotherapy (47–50). In this study, we observed prominent cytoplasmic and nuclear staining for SGK-1 while pSGK-1 staining was observed for cytoplasm and plasma membrane in hyperkeratotic oral tissue specimens. Interestingly, however, HPV-OPSCC specimens displayed mild and patchy cytoplasmic and nuclear staining. In our recent study with dysplastic and malignant (i.e., HPV-negative) oral tissue specimens, we also showed that while SGK-1 is primarily localized within the cytoplasm, pSGK-1 displayed cell membrane localization (7). While the reason for the localization pattern for the two forms of SGK-1 is not clear, it is noteworthy that perinuclear compartment is suggested to be the site of mTORC2-dependent phosphorylation of Ser422 of SGK-1, vs. Akt phosphorylation occurring at the plasma membrane, in HEK293 and opossum kidney tubule proximal cell lines (51). Thus, it is likely that SGK-1 phosphorylation is context- and cell-specific. Importantly, HPV-OPSCC specimens displayed marked reduction in both area of staining and normalized staining for SGK-1 and pSGK-1 compared to non-neoplastic tissue samples. This observation is similar to our recent finding of reduced levels of both SGK-1 and pSGK-1 in squamous cell carcinoma specimens (i.e., HPV-negative) compared to benign keratosis. Since pSGK-1 is the active and the pro-survival form of SGK-1, its reduction in is suggestive of an adaptive mechanism to curtail tumor growth, an aspect of translational relevance in relation to use of SGK-1 inhibitors as therapeutic options in cancer (47).

One limitation of our studies relates to a relatively small number of cases available in our archive for this investigation. However, it is unlikely that our observations regarding cellular localization of each protein of interest would be different with larger sample size and that semi-quantitative analyses revealed significant differentials between groups. The other limitation relates to very limited clinical pathological information (e.g., tumor stage) available to us because these patients presented to clinic for initial assessment when biopsy specimens were obtained before definitive treatment was rendered.

In conclusion, our results indicate differential localization and expression of GILZ, Annexin-A1, SGK-1 and pSGK-1, in human HPV-OPSCC. Given the complexities of these glucocorticoid-inducible proteins in tumor biology as well as the existence of a glucocorticoid system in oral mucosa, elucidation of contribution of each protein to pathogenesis of HPV-OPSCC is of clinical relevance and importance.

### Perspective

HPV encodes E6 and E7 oncoproteins which bind to p53 and retinoblastoma proteins, respectively, thereby eliminating their tumor suppressor protective functions resulting in uncontrolled cellular proliferation and tumorigenesis. While E6 causes accelerated ubiquitin-mediated degradation of p53, E7 binds to and sequesters retinoblastoma protein (4). In relation to tumorigenicity, we are not aware of the impact and/or interaction of GILZ or SGK-1 with either E6 or E7; however, Annexin-A1 is implicated in HPV cervical carcinoma via a mechanism related to preferential E6-mediated degradation of p53 compared to Annexin-A1 (52). Nonetheless, evidence exists for glucocorticoid-inducible proteins to influence tumorigenicity (13–15) and aspects related to p53 and/or retinoblastoma proteins may be pertinent to HPV-related OPSCC. For example, in colorectal carcinoma cells, a variant of GILZ, known as long GILZ, binds to p53 and mouse double minute 2 homolog (MDM2) thereby causing dissociation of p53/MDM2 complex and releasing p53 to exert its tumor suppressor function; MDM2 is the negative regulator of p53 due to formation of MDM2/p53 complex and ultimate induction of ubiquitin-mediated p53 proteosomal degradation (20). In addition, in thymus cells, GILZ mediates antiproliferative effect of glucocorticoids by inhibiting Ras-mediated signaling involving phosphorylation/inactivation of retinoblastoma, among other effects (53). On the other hand, based on studies pertinent to colon cancer, one aspect of the prosurvival activity of SGK-1 relates to phosphorylation of MDM2 and facilitation of p53/MDM2 complex formation with subsequent p53 proteosomal degradation (54). With respect to Annexin-A1, pancreatic tumors with high expression of this protein exhibited p53 mutation, epithelial-mesenchymal transition and worse outcomes (55). However, we are not aware of studies related to retinoblastoma protein for either SGK-1 or Annexin-A1. Clearly, there is a paucity of information regarding the role of glucocorticoid-inducible proteins, in the context of E6/, E7, p53 and retinoblastoma proteins, in HPV-related OPSCC and extrapolation of our findings to such mechanisms is far-reaching at this time. Nonetheless, our observations provide baseline information for subsequent investigation of their roles in pathogenesis of this condition, which, in turn, should contribute to appreciation of their diagnostic and/or prognostic potentials as well as whether they can serve as novel targets of therapy.

## Data Availability

The raw data supporting the conclusions of this article will be made available by the authors, without undue reservation.
